# A Comprehensive Review on Postpartum Depression

**DOI:** 10.7759/cureus.32745

**Published:** 2022-12-20

**Authors:** Om Suryawanshi, Sandhya Pajai

**Affiliations:** 1 Obstetrics and Gynecology, Jawaharlal Nehru Medical College, Datta Meghe Institute of Medical Sciences, Wardha, IND

**Keywords:** methyldopa, psychological, mental health, antidepressants, postpartum depression

## Abstract

One of the most common psychological effects following childbirth is postpartum depression. Postpartum depression (PPD) has a significant negative impact on the child's emotional, mental as well as intellectual development if left untreated, which can later have long-term complications. Later in life, it also results in the mother developing obsessive-compulsive disorder and anxiety. Many psychological risk factors are linked with PPD. The pathophysiology of the development of PPD is explained by different models like biological, psychological, integrated, and evolutionary models, which relate the result of the condition with particular conditions and factors. This article also explains the role of methyldopa as a medication used during pregnancy and the postpartum phase with the development of PPD. There are different mechanisms by which methyldopa causes depression. The large-scale screening of the condition can be done by Edinburgh Postnatal Depression Scale (EPDS). The diagnosis can be made by clinical assessment, simple self-report instruments, and questionnaires provided to mothers. Currently, there has not been any specific treatment for PPD, but selective serotonin reuptake inhibitors (SSRIs) like sertraline are effective in acute management. Venlafaxine and desvenlafaxine are serotonin-norepinephrine reuptake inhibitors used for the relief of symptoms. The SSRI and tricyclic antidepressants (TCA) used in combination have a prophylactic role in PPD. Nowadays, women prefer psychological therapies, complementary health practices, and neuromodulatory interventions like electroconvulsive therapy more than previous pharmacological treatments of depression. Allopregnanolone drug made into sterile solution brexanolone leads to a rapid decline of PPD symptoms. PPD is a common and severe disorder that affects many mothers following childbirth but is ignored and not given much importance. Later it affects the child's psychological and intellectual abilities and mother-child bonding. We can easily prevent it by early diagnosis and timely care and management of the mother. Understanding the underlying pathophysiology would also go a long way in preventing and managing the disorder.

## Introduction and background

Postpartum depression (PPD) is a significant mental health constraint in females, which has an effect on nearly 13-19% of the females who newly attained motherhood [1]. PPD is identified by a continuous feeling of a low state of mind in new mothers, followed by sad feelings, less worthy, and despondence. It differs from baby blues, a short-lived period of emotional disruption that includes weeping, irritability, sleep troubles, and anxiety. It is identified and felt by every four in five women in very few days after child delivery and mostly remits by 10 days [1,2]. Currently, the Diagnostic and Statistical Manual for Mental Disorders-Fifth Edition (DSM-5) has classified depression associated with the onset of childbirth as starting in pregnancy or by the first month of postpartum [3]. According to the International Classification of Disease (ICD), postpartum depression is labeled as one beginning by the first six weeks of the postpartum phase [4]. Many research studies have further revised the guidelines for the first six months following childbirth, while few use the time frame for up to the first year following the period of delivery for the beginning of PPD [5].

In most aspects, PPD has many likely features with depression which occur at other times in a mother's life; in some prospects, it has few differences as many significant changes occur during pregnancy and the postpartum phase [1,6]. In approximation, nearly 80% of postpartum women face the prodrome of emotional disturbances in the first few days following childbirth [7]. More of, a large proportion of postpartum women following pregnancy experience symptoms attributed to depression-like disturbed appetite, lack of sleep, and low energy levels for working [8]. The above factors make it hard to separately identify the commonly occurring symptoms following childbirth and new infant care from those of a depressive condition. Sometimes the phase of postpartum depression in nearly 30% of women can continue for two years postpartum [9], while 50% of women have major depression throughout in which the course of depression may vary and have stable moderate depression, major stable depression, or repetitive intervals of significant depression [10]. A comparison of symptoms between postpartum blues, postpartum depression, and postpartum psychosis is shown in Table 1.

**Table 1:** Comparison of symptoms between postpartum blues, postpartum depression, and postpartum psychosis. The table is adapted from Fishbein (2017) (Open source) [11].

Postpartum blues	Postpartum depression	Postpartum psychosis
Affects up to 80% of new mothers	Affects between 10% and 15% of new mothers	Affects one to two out of 1000 new mothers
Crying	Persistent sadness	Auditory and visual hallucinations
Sadness	Poor concentration	Paranoia
Anxiety	Feelings of worthlessness and guilt	Anxiety
Irritability	Irritability	Agitation
Insomnia	Anhedonia	Insomnia
Mood lability	Insomnia	Suicidal or homicidal thoughts
	Hypersomnia	Bizarre delusions or commands to harm the infant
	Somatic symptoms, poor bonding with infant, recurrent thoughts of death, or suicide	-

Psychological risk factors of PPD

**Figure 1:**
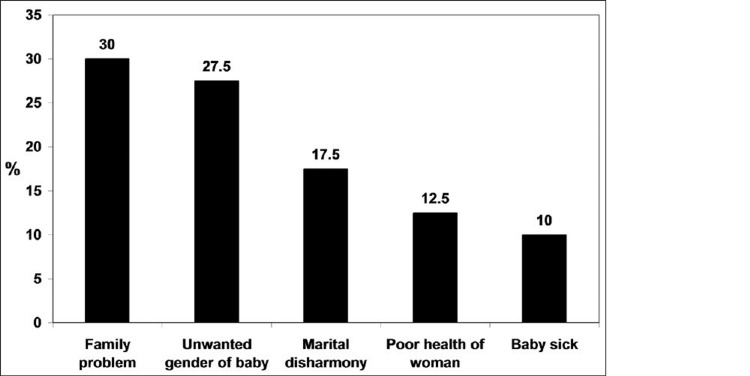
Factors attributed to postpartum depression. The image is adapted from Shriraam et al. (2019) (Open source) [20].

The risk factors can be grouped based on the strength of association with PPD. Depression and anxiety in pregnancy, postpartum blues, history of depression, neuroticism, excessive stress indulging life events, poor marital relations, lack of social support, and low self-esteem are strongly associated with postpartum depression [12]. On the other side, low socioeconomic status, single marital status, unwanted pregnancy, obstetrical stressors, and grieving infant temperament are reported to have a relatively weaker association [13,14]. The attitude of a mother [15] and her experience of different related complications like preterm delivery, prenatal hospitalization, emergency cesarean section, preeclampsia, and deceased infant health [16] are shown to have increased risk of developing PPD [17-19]. The above risk factors are more strongly associated with social and psychological aspects than biological aspects. A bar chart showing percentage of factors attributed to PPD is shown in Figure 1.

## Review

Pathophysiology of PPD

The exact mechanism for the development of postpartum depression is still unknown. There are many different models and theories explaining the condition's cause over time. Biological model explains the development of the condition due to the drastic and sudden decrease in many pregnancy hormones like progesterone, estradiol, and cortisol. In the withdrawal model, stress and reproductive hormones increase in pregnancy and fall drastically during childbirth and in the postpartum phase, which leads to dysregulation of the system and causes PPD [21-23]. However, they fail to explain the mechanism of hormone withdrawal with depression and the depressive symptoms which begin during pregnancy. The depression model states the association of PPD with the stress hormones dysregulation, mainly cortisol [24]. There is a suggestion from a few recent reviews for the role of dysregulation of the hypothalamic-pituitary axis in the causation of PPD [25,26]. Declined dopaminergic regulation may also have a role in PPD [27]. Many neuroendocrine changes in pregnancy can also affect PPD development, including inhibited Gamma-aminobutyric acid (GABA) signaling and low levels of allopregnanolone [28-30].

Psychological models focus mainly on the effect of pregnancy, childbirth, and new parenthood as the major stress factors which cause PPD symptoms in women. There has been much support in the psychological literature [31]. Integrated models bridge the above and state the role of both biological factors, such as stress causing PPD symptoms in women having genetic and hormonal susceptibility [23]. Evolutionary models have an evolutionary perspective in which the PPD is believed to be due to human civilization because of psychological adaptation in the course of human evolution. A "mismatch hypothesis" of PPD was recently proposed by Hahn-Holbrook and Haselton that suggests that PPD might be a "disease of civilization" due to significant cultural shifts over the past century that have resulted in substantial deviations from typical human evolutionary lifestyles and the current high incidence rate [32].

Role of methyldopa in the induction of postpartum depression

Methyldopa, an agonist of presynaptic alpha-2 adrenergic receptors, prevents neurons from releasing norepinephrine and, consequently, inhibits the sympathetic nervous system. This medication is actively transported to the brain as an amino acid, where it is metabolized into the active form, -methyl norepinephrine. In the biosynthesis pathway of dopamine, norepinephrine, and epinephrine, methyldopa replaces dihydroxyphenylalanine (DOPA), forming inactive structures of neurotransmitters. Methyldopa blocks the baroreceptor signaling pathway by activating presynaptic 2-adrenergic receptors and altering a single nucleus through inactive neurotransmitters [33].

Methyldopa significantly raises the level of vascular endothelial growth factor (VEGF), which is both an angiogenic factor and a neurotrophic agent. Although neuronal function alteration is probably more complex, VEGF dysfunctions neurogenesis and the functioning of sustained neurons by decreasing serotonin concentration and catecholamine levels due to this property. These changes characterize the neurotrophic depression model.

Methyldopa reduces cerebral blood flow by impairing baroreceptor signaling pathways and decreasing sympathetic system stimulation. Impaired neuronal function, cognitive decline, and depression are all consequences of decreased cerebral blood flow, particularly in the orbitofrontal cortex. These modifications characterize the vascular model of depression.

Methyldopa raises nitric oxide (NO) levels by reducing nitric-compound excretion in the kidneys and increasing endothelial nitric oxide synthase (eNOS) expression. In high concentrations, NO is neurotoxic, causing mild inflammation and decreased levels of cofactors (tryptophan, tetrahydrobiopterin, and others).and decreased levels of catecholamines and serotonin; consequently, elevated NO levels can cause depression. Methyldopa lowers dopamine concentration by interfering with its production. The excretion of prolactin is controlled by dopamine. Low dopamine levels cause hyperprolactinemia, which impairs sexual behavior and contributes to depression. Disruption of the reward system is an integral part of the development of depression. Methyldopa lowers dopamine levels, a neurotransmitter essential to the reward system. Depression is brought on by methyl-dopa through this mechanism.

In light of the preceding, taking methyldopa can cause depression. Because methyl-dopa is the first-line treatment for preeclampsia and hypertension in pregnancy and because mood swings and sluggishness are common after labor, this side effect of methyldopa is more likely to occur in pregnant women. To fully understand the problem and provide appropriate mental health care for patients, extensive prospective studies evaluating depression that occurs during the treatment of methyldopa and identifying potential prevention and treatment are required in light of the solid theoretical foundation.

**Figure 2:**
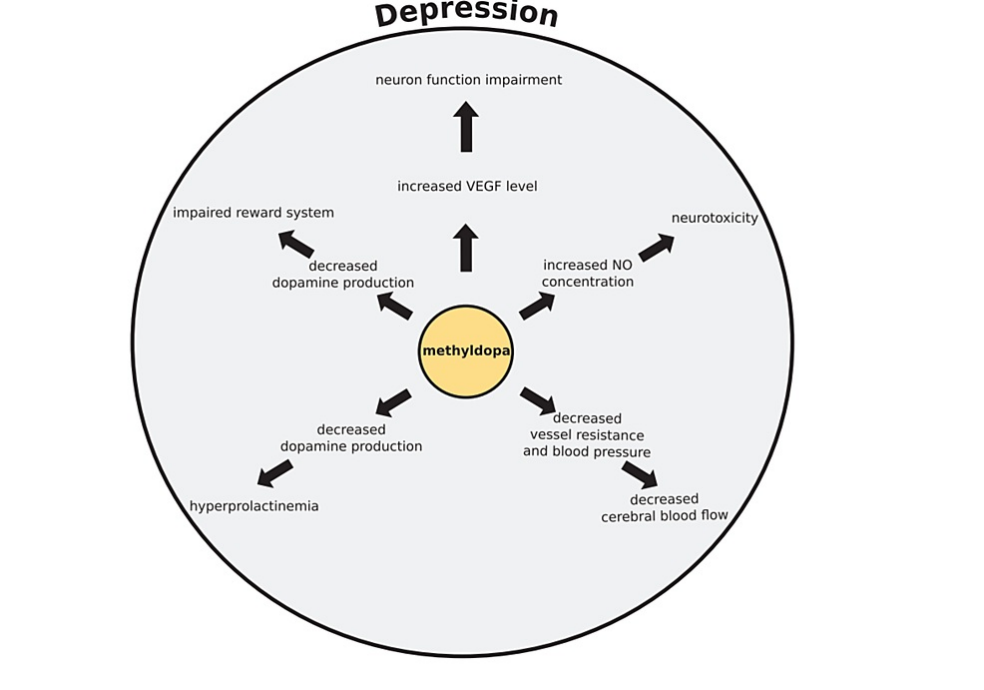
Mechanism of depression induction by methyldopa. The image is adapted from Wicinski et al. (2020) (CC BY 4.0) [33].

Methyldopa may be considered a depression risk factor, inducer in postpartum depression, with the cause of maternal blues in light of the preceding data. This process's pathomechanism is intricate and classified into following five categories: (1) neurotrophic alteration, (2) reduction of cerebral blood flow, (3) neurotoxicity induced by NO, (4) high levels of prolactin, and (5) reward system impairment (Figure 2).

Diagnosis of PPD

The criteria for when PPD first appears is still debatable [34]. The United States Diagnostic and Statistical Manual of Mental Disorders-Fifth Edition (DSM-5) includes episodes that begin during pregnancy and last for six months after birth [35]. Postpartum depression (PPD) has been estimated to occur up to one year after childbirth in clinical practice and published research. For example, DSM-IV’s Structured Clinical Interview can be used to diagnose PPD Simple self-report instruments like questionnaires have been used for many clinical assessments. The Edinburgh Postnatal Depression Scale (EPDS) is reliable, validated, frequently more practical, and economical in large-scale screenings for PPD risk and is the most well-known and widely used [36]. To lessen the burden placed on common symptoms experienced by most new mothers, EPDS emphasizes psychic depression symptoms. Two-item Patient Health Questionnaire (PHQ-2) and nine-item Patient Health Questionnaire (PHQ-9) questionnaire-based screening tools are familiar. The first two items of the PHQ-9 can be found in PHQ-2. A typical score of 10 or higher on the EPDS or PHQ-9 [37-40] is used as the threshold for being positive for PPD Many brief E.P.D.S. subscales, including three-item, seven-item, and -item subscales, have also been developed [41]. The Hamilton Rating Scale for Depression (HAM-D), which isn’t explicitly made for PPD, is one of the other screening tools [42]. HAM-D's reliability varies significantly between 0.46 and 0.98 in various evaluations [43]. The Bipolar Spectrum Diagnostic Scale (BSDS) for bipolar disorders (BD) and other scales for diagnosis of related mood disorders may also be functional in the perinatal period [44].

Present-day treatment options of PPD and its constraints

There are many therapeutic interventions in the treatment of PPD, most adapted from the treatment of the major depressive disorder (MDD), as to date, there aren't any pharmacological therapies explicitly approved for PPD

Acute Treatment

Selective serotonin reuptake inhibitors (SSRI): The primitive line treatment for moderate to severe PPD by use of an SSRI. Among all clinical trial drugs, Sertraline is the most effective drug among SSRIs for treating PPD [45]. De Crescenzo and colleagues conducted a systematic review in which they found psychotherapy, SSRIs, and Nortriptyline are adequate for the acute treatment of PPD [46]. However, insufficiently proven studies clearly distinguish one remedy from another.

Serotonin norepinephrine reuptake inhibitors (SNRIs) and antidepressants: There hasn't been much-randomized control trials (RCT) trial data for SNRIs and antidepressants. Open-label trials recommend venlafaxine [47] and desvenlafaxine [48] to relieve symptoms.

Tricyclic antidepressants (TCA) and monoamine oxidase inhibitors (MAOI): Till today, nortriptyline is one TCA used for PPD [49]. There are not much RCT-level data for MAOI.

Prophylactic Treatment

Selective serotonin reuptake inhibitors (SSRIs) and tricyclic antidepressants (TCA's): The risk of recurrence for women who have previously experienced PPD is approximately 25% [50]. To conclude the efficacy of antidepressants in preventing PPD, a newly published Cochrane review found that additional studies involving more participants are required [51].

Psychotherapies, Complementary Health Practices, and Neuromodulatory Interventions

When considering antidepressant treatment, women having PPD experience mild to high rates of decisional conflict, particularly during pregnancy [52], and many prefer psychotherapies to pharmacotherapies [53]. Nearly 26-75% of pregnant women worldwide use complementary health practices due to their significant health-related advantages [54]. In the United States, 54% of women who suffer from depression say they have used complementary health practices in the past year [55]. In more severe and remitting cases of postpartum depression (PPD) and postpartum psychosis, an important neuromodulatory option is electroconvulsive therapy (ECT). Compared to treatment for non-postpartum depression or psychosis, ECT is said to have a higher response rate [56]. There are no RCT-level data for using ECT to treat PPD, despite the publication of guidelines for its use during Pregnancy [57]. For the treatment of PPD, additional neuromodulatory methods, such as transcranial direct current stimulation (TCCS) and repetitive transcranial magnetic stimulation (TMS), are in their initial trial stages [58-62]. Other non-invasive neuromodulation interventions and the effectiveness of ECT versus pharmacotherapy in severe PPD should be subject to additional RCT.

Implications for novel pharmacological treatment

A clinical study has used intravenous preparations for endogenous allopregnanolone CNS drugs due to its less oral bioavailability and excessive in vivo clearance. Allopregnanolone given intravenously has been shown to cause sedation and decreased saccadic eye velocity, with women experiencing these effects more than men [63]. Some women who receive intravenous allopregnanolone may experience only episodic and not semantic or working memory impairment [64]. Acute intravenous administration of allopregnanolone does not affect the startle response or prepulse inhibition of the startle reaction, indicating that it does not have anxiolytic effects on healthy women [65]. Allopregnanolone may regulate the hypothalamic-pituitary-gonadal axis through GABA-A receptor modulation, as evidenced by the fact that intravenous administration in healthy women during the follicular phase of a menstrual cycle is linked with decreased plasma concentration of luteinizing hormone and follicle-stimulating hormone but not plasma levels of estradiol or progesterone [66]. Investigation of synthetic Non-allogenic steroids (NAS) and their analogs as primary treatments for PPD is supported by the evidence mentioned above that NAS and GABA play a role in the pathophysiology of PPD Brexanolone (USAN) was developed by Sage Therapeutics, formerly known as SAGE-547 Injection), a proprietary, soluble synthetic allopregnanolone intravenous preparation. Brexanolone, which is a sterile solution of 5 mg/mL allopregnanolone in 250 mg/mL sulfobutylether-cyclodextrin buffered with citrate and diluted until it is isotonic with sterile water [67]. Brexanolone causes potent, dose-dependent activation of GABA-mediated currents in whole-cell patch electrophysiology studies. Studies on drug interactions have shown that co-administration of brexanolone can alter the metabolic rate of CYP2C9 substrates. Brexanolone in PPD was the subject of the latest series of open-label and few RCTs, which are placebo-controlled that demonstrated a rapid decline in PPD symptoms.

Consequences of PPD

There are few denotations related to women who experience postpartum depression being more likely to have comorbid obsessive-compulsive disorder and anxiety than women who experience depression at other times in their lives [68]. Postpartum depression is linked to various outcomes in other areas and an increased risk of comorbid disorders [69]. There have been reports of adverse long-term effects on infants' social, emotional, intellectual, and physical development [70]. Postpartum depression-afflicted mothers' children may also be more likely to have intellectual disabilities and psychosocial, emotional, or behavioral problems [71]. Deficient parenting and parental safety practices are also linked to postpartum depression and difficulties in bonding and mother-child interactions. Research must identify the significant risk factors and protective factors for postpartum depression because of the potentially devastating effects on the mother, the child, and their family postpartum depression.

## Conclusions

PPD is a disorder that can be crippling and common. There are several effective pharmacological therapies, psychological therapies, psychosocial, and neuromodulation intercession, but the majority are understudied, particularly in RCT. Sadly, there is a significant underutilization of available treatments in the community. Even though PPD is now more readily discussed, a considerable stigma exists against few women seeking treatment. In low socioeconomic countries, mental health might not be prioritized; women may have restrictions in reaching out to providers specially trained in perinatal mental health even when they seek treatment. Because of the complexity of treatment modalities of peripartum psychiatric illness demands integrative work among multiple health service providers, including obstetrics, psychiatry, pediatrics, and nursing/midwifery. Reproductive psychiatric tutorials should be spread widely within the discipline of psychiatry in residency and fellowship programs.

It is necessary to develop novel therapeutics that specifically target the disorder's underlying pathophysiology and expand access to the treatments that are already in place and improve the quality of those treatments. The underlying neurobiology of PPD is still poorly understood, despite increased research into its causes. There is mounting evidence that psychiatric disorders are neural network disorders characterized by complex, multimodal patterns of neurobiological abnormalities. As a result, there is a pressing need for additional research into the underlying mechanisms of these disorders. We can detect, diagnose, and treat PPD more effectively during pregnancy and postpartum if we learn more about the neurobiology of PPD.
